# 2020s Heroes Are Not Fearless: The Impact of the COVID-19 Pandemic on Wellbeing and Emotions of Italian Health Care Workers During Italy Phase 1

**DOI:** 10.3389/fpsyg.2020.588762

**Published:** 2020-10-15

**Authors:** Giulia Marton, Laura Vergani, Ketti Mazzocco, Marina Chiara Garassino, Gabriella Pravettoni

**Affiliations:** ^1^Applied Research Division for Cognitive and Psychological Science, IEO, European Institute of Oncology IRCCS, Milan, Italy; ^2^Department of Oncology and Hemato-Oncology, University of Milan, Milan, Italy; ^3^Thoracic Oncology Unit, Division of Medical Oncology, Foundation IRCCS–Italian National Cancer Institute, Milan, Italy

**Keywords:** health care workers, COVID-19, emotional reaction, health care workers wellbeing, distress

## Abstract

**Objective:**

The study aimed to investigate the mental health and emotional reaction of physicians working during phase 1 of the COVID-19 pandemic in Italy.

**Methods:**

A total of 458 Italian Health Care Workers (HCWs) working during phases 1 of the COVID-19 outbreak were voluntarily enlisted in the study and recruited with the snowball technique through an online survey. We examined our variables with the General Health Questionnaire – 12 and with Visual Analog Scales.

**Results:**

The sample has a high level of psychological distress 21.26 (SD = 4.46), the emotional reaction was characterized by high level of fear for family members and cohabitants (*M* = 77.67, SD = 27.16) and patients (*M* = 67.16, SD = 27.71). Perceived control, fear for patients, and for family members and cohabitants, feeling alone and anger all contribute to a decreased mental health in Italian physicians (*R*^2^ = 0.285, p < 0.001).

**Conclusion:**

Italian HCWs’ mental health and emotional reaction have to be considered to prevent high risk of burnout and post-traumatic stress disorder (PTSD). It becomes pivotal in the next months to implement a tailored psychological intervention to take care of HCWs and to prevent costly consequences for them, patients, and the healthcare system.

## Introduction

On the 11th of March 2020, the World Health Organization characterized COVID-19 as a pandemic. In Italy the situation was already very serious and, as one of the first States to be affected by this outbreak, the spread of the disease was at its peak, with the national lockdown imposed on the 9th of March. Only on the 18th of May phase 1 ended, with the restrictive measure of lockdown loosen and the healthcare system registering a break from the emergency. COVID-19 spreads rapidly and can cause severe symptoms, giving a lot of pressure to the National Health System (Shanafelt et al., 2020). Health care workers’ (HCW) workload was very demanding, affected also by suspension of days of leave and rest. Moreover, the strict contact with infected patients and the risk of being infected themselves increase the physical and psychological difficulties that HCWs have to face: the fear is not limited to the possibility to get the virus, but also to the possibility to take it home and infect their families and other people as proven during other pandemics in the past (Maunder et al., 2006). At the beginning of July 2020, more than 1800 HCWs have died because of the COVID-19 (MEDSCAPE, 2020). Also, the limited number of beds in the intensive care units and the dramatically increased number of patients needing intubation, imposed HCWs the responsibility to choose which patients to cure (Rosenbaum, 2020). Such a dilemma accumulated with the aforementioned factors of psychological distress. Studies conducted in past comparable situations showed that the stressful situation, the workload, and the high responsibility affect the psychological wellbeing of HCWs, with acute and chronic consequences (Bai et al., 2004; Chen et al., 2005; Maunder et al., 2006; Khalid et al., 2016; Lai et al., 2020; Li et al., 2020; Zhu et al., 2020). Post traumatic stress, insomnia, depressive, and anxiety symptoms are often reported by HCWs during pandemic and epidemic situations (Preti et al., 2020). During the SARS epidemic, quarantine was associated with emotional distress in HCWs as well as feelings of fear to contract the disease, worry for the family and isolation, the stress in the workspace and stigma of possibly being contagious (Bai et al., 2004; Maunder et al., 2006). Under these conditions, working in a hospital that treated SARS patients led to high burnout levels, psychological distress and post-traumatic stress disorder (PTSD) (Chen et al., 2005). During the MERS-CoV outbreak in 2014, HCWs experienced fear for personal safety and fear for their colleagues and families (Khalid et al., 2016). In China, the first country to be affected by COVID-19, HCWs working with COVID-19 patients reported various symptoms of psychological distress, like anxiety, insomnia and depression (Huang and Zhao, 2020; Lai et al., 2020; Zhu et al., 2020). A preliminary study on trauma during the COVID-19 pandemic (Li et al., 2020) suggested that HCW may experience vicarious traumatization due to the frequent experience of seeing patients dying without having their loved-ones near them.

But what are the main factors that affect the psychological distress of HCWs during COVID-19 pandemic? The aim of this study was to explore the mental health and emotional reactions of Italian HCW involved in phase 1 of the COVID-19 pandemic. Based on previous studies conducted during similar events, we hypothesized a decreased mental health and emotional distress affecting Italian HCW during phase 1 of the COVID-19 pandemic.

## Methods

For this observational study, we recruited a sample of 458 HCW working during the first phases of the COVID-19 Italian outbreak, through HCWs mailing lists, social media and snowball recruitment. For the considered population, a minimum sample of 400 respondents allows having a certainty measure with a confidence level of 95% and a confidence interval of 5% (Hill, 1998). Recruitment started on the 24th of March until the 13th of May and focused on phase 1 of the COVID-19 Italian emergency.

The sample comprised HCWs working all over Italy during the Pandemic, with a mean age of 43.46 years (SD = 10.22; range: 25–70 years), a mean of 15.03 (SD = 10.23) years of working experience, and a mean of 36.30 (SD = 17.73) hours of work per week, with an average of 1.63 days of rest per week (SD = 0.84).

Our sample was composed by 79% (362 out of 458) physicians, 9.8% (45 out of 458) nurses, 5.2% (24 out of 458) technicians, 1.7% (8 out of 458) psychologists, 0.7% (3 out of 458) OSS, 0.7% (3 out of 458) OTA, 0.7% (3 out of 458) volunteers, 0.4% (2 out of 458) pharmacists, 0.2% (1 out of 458) obstetricians and 1.5% (7 out of 458) other kind of HCWs.

The study was approved by the European Institute of Oncology ethics committee (R1185/20-IEO 1248). Participants provided written informed consent before being asked to fill in an online survey characterized by a standardized questionnaire to measure the mental health status (the 12-item General Health Questionnaire, GHQ; Goldberg et al., 1997), and Visual Analog Scales (VAS) to assess personal experience associated with the situation. In particular, general distress, fear for themselves, their family members and cohabitants and their patients, the anger felt in this period, the perceived level of loneliness and the perceived level of abandonment by the Institutions were assessed. Given the association between perception of control on the situation and the presence of distress (Bhanji et al., 2016), perceived control on the situation was also measured with a VAS. The 12-item GHQ was characterized by 4-point Likert scale answers, with low scores indicating a good mental health status and high scores indicating a bad mental health status. Scores above the threshold of 13/14 indicate the presence of psychological distress (Piccinelli et al., 1993; Goldberg et al., 1997). The VAS were on a range from 0 (not at all) to 100 (completely), with higher values indicating a worse condition except perceived control of the situation. Finally, questions were asked on workload-related information (average number of rest days per week in this period and average working hours per week in this period) and socio-demographic information (years of working experience and age).

We performed descriptive statistics (mean and standard deviation) and, considering a statistical significance of *p* > 0.05, we performed a bivariate correlational and a stepwise backward regression analysis on collected data. We performed our analysis with SPSS 26.

## Results

Descriptive statistics for GHQ-12 scores and Visual Analog Scales for all 458 participants are reported in Table 1. The mean score for GHQ-12 was 21.26 (SD = 4.46), indicating a generally high level of psychological distress. The high score in psychological dysfunction was confirmed by physicians’ perceived distress directly measured with VAS (*M* = 67.90, SD = 23.16, *r* = 0.54, *p* < 0.01).

**TABLE 1:** Variables descriptive statistics and correlations.

		Mean	SD	*N*	1	2	3	4	5	6	7	8	9	10	11	12	13
1	GHQ-12	21.26	4.466	458		0.540**	0.311**	0.359**	0.339**	−0.225**	0.369**	0.382**	0.330**	−0.064	0.042	−0.029	0.008
2	Perceived stress	67.9	23.161	458			0.473**	0.439**	0.437**	−0.179**	0.404**	0.392**	0.386**	−0.132**	−0.014	−0.001	0.036
3	Fear for themselves	48.59	30.989	458				0.464**	0.258**	−0.142**	0.348**	0.291**	0.379**	−0.016	−0.079	0.074	0.115*
4	Fear for family members/cohabitants	77.67	27.16	458					0.492**	−0.150**	0.309**	0.238**	0.355**	−0.068	−0.006	−0.055	−0.015
5	Fear for patients	67.16	27.713	458						−0.134**	0.250**	0.215**	0.330**	−0.021	0.018	−0.018	0.002
6	Perceived control of situation	41.21	26.851	458							−0.140**	−0.168**	−0.158**	−0.051	0.015	0.120*	0.110*
7	Anger	48.62	34.625	458								0.401**	0.489**	−0.01	−0.057	0.047	0.076
8	Feeling alone	47.56	34.727	458									0.442**	−0.082	−0.017	0.004	0.049
9	Feeling abandoned by institutions	61.15	32.593	458										0.027	−0.036	0.031	0.055
10	Days of rest per week	1.637	0.8482	452											−0.223**	−0.015	−0.045
11	Hours of work per week	36.304	17.7377	455												0.021	0.041
12	Years of working experience	15.031	10.2339	458													0.921**
13	Age	43.4639	10.22828	457													

*Significant correlations 0.05 two-tailed; **significant correlations 0.01 two-tailed.

Regarding specific emotional reactions due to the COVID-19 emergency, participants indicated high levels of fear for family members and cohabitants (*M* = 77.67, SD = 27.16) and fear for patients (*M* = 67.16, SD = 27.71). Instead, fear for themselves received a significantly lower score (*M* = 48.59, SD = 30.98) compared to the experience of fear for others – fears for family members and cohabitants and for patients [respectively *t*(457) = −20.55, *p* < 0.001 and *t*(457) = −11.08, *p* < 0.001); moreover, fear for family members was significantly higher than fear for patients [*t*(457) = 8.14, *p* < 0.001]. Perceived control related to the situation was the lowest score (*M* = 41.21, SD = 26.85). Correlation analysis demonstrated a significant negative association between perceived control of the situation and emotional reactions (*p* < 0.01). A negative correlation was found also between perceived control and general mental health (*p* < 0.01) and general distress (*p* < 0.01).

A stepwise backward regression analysis was performed to analyze the predictive effect of fear (for themselves, for family members and cohabitants and for patients), anger, feeling alone, perception to feel abandoned and perceived control on mental health. The final model included perceived control, fear for patients and family, feeling alone and anger as significant predicting factors, explaining 28.5% of the variability in the total GHQ-12 score (*R*^2^ = 0.285, *p* < 0.001). Of these variables, feeling alone significantly made the largest contribution in the GHQ-12 score (β = 0.221), followed by anger and fear for family members and cohabitants, fear for patients, and perceived control of the situation (respectively: β = 0.176; β = 0.159; β = 0.153; β =−0.119).

Regression analysis showed also a predictive role of years of experience, feeling alone, fear for patients, and fear for themselves on perceived control (*R*^2^ = 0.062, *p* < 0.001), with a lower perceived control in presence of higher levels of feeling alone (β = −0.117), fear for patients (β = −0.081) and fear for themselves (β = −0.099). On the contrary, years of working experience significantly affected the perceived control (β = 0.133, *p* < 0.05), with more years being associated with a higher perceived control.

## Discussion

During COVID-19 pandemic’s phase 1, HCWs’ life was surrounded by fear. Usually, HCWs are afraid of being blamed and punished but in this period a new fear is added (Gorini et al., 2012). They fear for their patients, and when they finish the endless time in the hospital, they go back to their home and they are afraid to infect families or cohabitants. The fear affects directly the mental health status, and also the perceived control on the situation that, in turn, influences the HCWs’ psychological state. Despite fear being a predictive factor of mental health and distress, feeling alone and anger emerged as relevant emotions affecting HCWs’ psychological wellbeing. Our results are in line with previous studies about these themes (Sadler and Weiss, 1975; Simard et al., 2013). The relevance of loneliness as a contributor to mental health is confirmed by previous studies showing its predictive role in the development and maintenance of depressive and anxiety symptoms (Wang et al., 2018; Hill and Hamm, 2019). Moreover, loneliness has been found to have impact on other chronic and various diseases: for example, cognitive decline (Shankar et al., 2013), cardiovascular diseases (Herlitz et al., 1998; Sorkin et al., 2002; Hawkley et al., 2010), cancer (Antoni et al., 2006), and inflammatory diseases (Luanaigh and Lawlor, 2008).

Our study links primary emotions with a cognitive aspect: the perception of lack of control. Considering the stress and negative emotions, together with the perceived difficulties in controlling the situation, it is not surprising that these findings are related to mental health.

In COVID-19’s phase 1, the pick of infected people was at its maximum and HCWs had to make rapid and often ethically challenging decisions on who and how to care (Wallace et al., 2020). Usually, professionals’ decision-making priorities should consider patient preferences (Marton et al., 2020; Monzani et al., 2020) to empower patients to reach the preferred decision (Arnaboldi et al., 2020). In this period, however, the low availability of ventilators compared to the high number of critical patients required HCWs to make life-or-death decisions (Rosenbaum, 2020). This might cause “*decision fatigue*,” a psychologically taxing phenomenon originating from the evaluation of pros and cons to make a good decision in the context of high potential risks (Baumeister et al., 1998) that, if not managed, will lead to higher distress (Chen et al., 2018). Moreover, to adapt to the complex environment, HCWs use heuristics that become inevitable (Mazzocco and Cherubini, 2010), and lead to mistakes. On top of that, preliminary studies on trauma during the COVID-19 pandemic (Li et al., 2020) suggested that HCWs may experience vicarious traumatization and emotional dissociation from what they are seeing and experiencing (Masiero et al., 2020): this may lead to PTSD if not timely managed. PTSD may have tremendous consequences and should be monitored not only in the patients’ population (Arnaboldi et al., 2014) but also in the HCWs’ one.

Perceived lack of control, high level of stress, negative emotions – all symptoms retrieved in our sample – are risk factors for Burnout Syndrome. Potential consequences of not managed distressed conditions will have implications not only for HCWs (Suñer-Soler et al., 2014) but also for patients and health systems: physical problems, diminished job satisfaction, less quality of care, absenteeism, negative attitude. It is fundamental, then, to implement actions and interventions to take care of physicians and HCWs during and after the emergency, to prevent a more costly situation.

The study presents some limitations. The sample only comprehends Italian HCWs, making the results not comprehensive of possible different findings of other nationalities. However, we think that the Italian experience remains an interesting context to consider. Another limit is that our model only explains 28.5% of HCW’s mental health. It is advisable that future research should consider other factors that could explain psychological distress in a more comprehensive way (i.e., individual aspects, the personality of the HCWs).

We also did not consider the working environment of the HCWs that could have caused a difference in how the COVID-19 outbreak has impacted the professionals. Some areas of Italy were more impacted than others and some hospital wards became COVID-19 specific. HCWs that worked in such highly impacted hospitals could have been more affected than others. In particular, we assume the different settings to have an impact on the decision fatigue experienced by the HCWs and on their emotional reactions that we mentioned above.

Among other results, we retrieved a detrimental emotional reaction of HCWs; moreover, perceived control, fear for patients and for families, feeling alone and anger, predict mental health. To mitigate these symptoms and to prevent their evolution in chronic diseases, it is pivotal to implement tailored psychological interventions that help HCWs to develop and improve skills in order to manage their emotional reactions, cope to the stressful working environment and foster their psychological well-being (Masiero et al., 2018).

## Data Availability Statement

The datasets presented in this article are available from the corresponding author on reasonable request. Requests to access the datasets should be directed to giulia.marton@ieo.it.

## Ethics Statement

The studies involving human participants were reviewed and approved by the European Institute of Oncology ethics committee (R1185/20-IEO 1248). The patients/participants provided their written informed consent to participate in this study.

## Author Contributions

GM, LV, and KM planned and conducted the study and drafted the manuscript. GP and MG supervised all the processes, provided critical guidance, and revised the manuscript. All authors contributed to the article and approved the submitted version.

## Conflict of Interest

The authors declare that the research was conducted in the absence of any commercial or financial relationships that could be construed as a potential conflict of interest.
